# Dinâmica integrada de controle cinético e cinemático na função de pinça da mão: Efeitos posturais e visual

**DOI:** 10.1055/s-0045-1810043

**Published:** 2025-08-18

**Authors:** Bernardo Figueira Althoff, João Carlos Nakamoto, Mateus Saito, Luiz Sorrenti, Ricardo Boso Escudero, Erick Yoshio Wataya

**Affiliations:** 1 Intituto Vita, São Paulo, SP, Brasil

**Keywords:** movimento, punho, retroalimentação, feedback, movement, wrist

## Abstract

**Objetivo:**

Interpretar, com dados objetivos, o controle cinético e cinemático na função da pinça associado ao controle visual e estereognóstico.

**Métodos:**

Ao todo, 34 participantes jovens, sem doenças ou traumas prévios nas mãos, foram submetidos à aplicação de tarefas com pinça, com o punho em posição neutra e fletida a 45°. As tarefas foram repetidas três vezes.

**Resultados:**

A diferença do movimento nas posturas neutra e flexionada a 45° foi significativamente correlacionada às variáveis de distância polpa-a-polpa (valores de r = entre 0,38 e 0,41; *p* < 0,05). A diferença da força nas posturas neutra e flexionada a 45°, com ou sem *feedback* visual, também apresentou correlação significativa (valores de r =entre 0,45 e 0,47; *p* < 0,01). A diferença do movimento foi significativamente correlacionada com a diferença da força na postura neutra sem *feedback* visual (r = 0,77; *p* < 0,001) e na postura flexionada com *feedback* visual (r = 0,48; *p* = 0,004).

**Conclusão:**

O controle de força e de movimento na função da pinça em adultos saudáveis está relacionado ao *feedback* visual e à postura do punho. Esses achados reforçam a interdependência dos mecanismos de controle na função manual. Ajustes posturais e o aprimoramento da propriocepção podem otimizar a recuperação funcional, com implicações para o desenvolvimento de testes específicos e sua aplicação em ambientes clínicos reais.

## Abstract (en)

**Objective:**

To interpret, with objective data, the kinetic and kinematic control of gripper function associated with visual and stereognostic control.

**Methods:**

In total, 34 young participants, without previous hand diseases or traumas, underwent pinch grip tasks with the wrist in a neutral position and at 45° of flexion. The tasks were repeated three times.

**Results:**

The movement difference in the neutral and 45° of flexion postures presented a significant correlation with the pulp-to-pulp distance variables (r values from 0.38 to 0.41; *p* < 0.05). The strength difference in the neutral and 45° of flexion postures, with or without visual feedback, also showed a significant correlation (r values from 0.45 to 0.47; *p* < 0.01). The movement and strength differences presented a significant correlation in the neutral posture without visual feedback (r = 0.77; *p* < 0.001) and in the flexed posture with visual feedback (r = 0.48; *p* = 0.004).

**Conclusion:**

Visual feedback and wrist posture influence strength and movement control in gripping function in healthy adults. These findings reinforce the interdependence of control mechanisms in hand function. Postural adjustments and proprioception enhancement can optimize functional recovery, with implications for the development of specific tests and their application in actual clinical settings.

## Introdução

A mão humana é um órgão complexo, constituído por ossos, músculos, tendões, fáscias, ligamentos, nervos e vasos sanguíneos envoltos por uma pele única dorsal frouxa e uma pele glabra palmar. É uma estrutura extremamente adaptável ao objeto e à tarefa, e representa anos de evolução. A capacidade de pinçar e manipular objetos é fundamental para o progresso da raça humana, e cujo destino foi moldado pela manipulação de ferramentas. 1 2 Do ponto de vista fisiológico, a mão é considerada a “extremidade realizadora”, pois permite adotar inúmeras posturas a fim de realizar diversas funções. Do ponto de vista cinético e cinemático, a mão humana apresenta uma complexidade funcional que lhe proporciona uma gama de possibilidades nas posturas, nos movimentos e nas ações. A função de pinça de preensão alcança graus não vistos em outros animais. Isso se deve à posição peculiar que apresenta o polegar, de poder opor-se a todos os outros dedos. 3 O objetivo deste trabalho é interpretar, com dados objetivos, o controle de pinça, seja na aplicação de força, seja na distância entre as polpas do polegar e do indicador durante a presença e ausência de *feedbacks* visuais e estereognósticos em diferentes posturas do punho. Tais informações podem trazer percepções para intervenções clínicas, reabilitação e *design* ergonômico.

## Materiais e Métodos

**Participantes:** foram incluídos participantes jovens de 20 a 40 anos, sem comorbidades, nem patologias conhecidas na mão. Foram excluídos indivíduos com alguma cirurgia ou procedimento prévio nas mãos, portadores de síndromes compressivas dos nervos periféricos do membro superior, artrites inflamatórias sistêmicas, portadores de artrose de articulações da mão e do punho, e pacientes que se recusaram a assinara o termo de consentimento livre e esclarecido. Este trabalho teve a aprovação do comitê de ética da nossa instituição sob o número CAAE: 66899023.2.0000.5474.

**Fig. 1:**
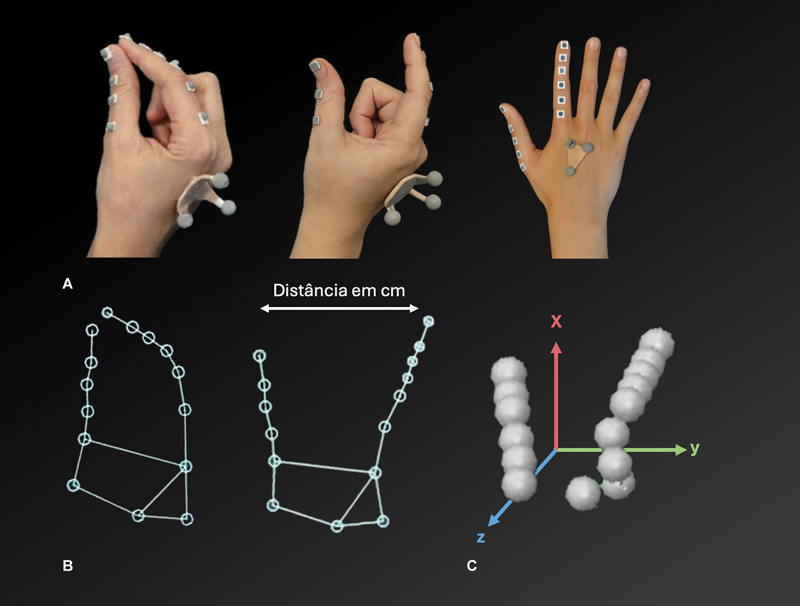
Coleta de dados de movimento: sistema de captura por marcadores passivos refletivos, registrados por câmeras infravermelhas posicionadas em volta da área de teste. ( **A** ) Posicionamento dos marcadores sobre o dorso da mão, o polegar e o indicador. ( **B** ) Reconstrução bidimensional da mão com marcação da distância polpa-a-polpa (em centímetros). ( **C** ) Reconstrução tridimensional com indicação dos eixos espaciais X (vermelho), Y (verde) e Z (azul).

**Coleta dados de movimento e distância polpa-a-polpa:** foi determinada a pinça da polpa do indicador com a do polegar. Foi utilizado um sistema com marcadores passivos capturados por câmeras infravermelhas posicionadas em volta da área de teste para coletar dados dos movimentos do polegar e do dedo indicador durante a tarefa de movimento de pinça longo dos eixos X (lateral), Y (vertical) e Z (profundidade) e medir a cinética tridimensional. Os marcadores refletivos foram colocados em pontos estratégicos (2 em cada falange tanto do polegar quanto do indicador), o que permitiu uma análise dos movimentos em três dimensões ( Fig. 1 ).

**Fig. 2:**
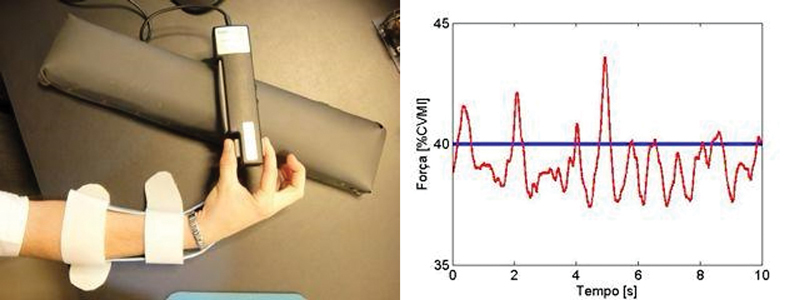
Coleta de dados de força: sistema de dinamometria para pinça com transdutor digital (MedEOR MedTech) acoplado a programa de medição.

**Coleta de dados de força polpa-a-polpa:** Foi usado um sistema de dinamômetro para pinça com transdutor digital (MedEOR MedTech) com programa que traduz em gráficos visuais o nível da força e do alvo em tempo real para medir a força durante a realização de pinça ( Fig. 2 ).

A variação da distância entre as polpas do polegar e do indicador nos fechamentos de pinça (distância polpa-a-polpa resultante e distância polpa-a-polpa em cada direção com máxima extensão do indicador e máxima extensão e abdução do polegar) e a variabilidade da força na tarefa de pinça isométrica foram descritas pelo desvio padrão de cada série temporal e usados como indicadores de desempenho durante as tarefas solicitadas.

## Testes

Ao todo, 34 participantes jovens, sem doenças ou traumas prévios na mão, foram submetidos à aplicação de tarefas com pinça. Foram solicitadas duas tarefas:

- Movimento de pinça de precisão: realizado com o polegar e o indicador, os participantes fizeram o movimento de pinça mantendo um ritmo de 2 segundos para cada ciclo de 25 segundos. A tarefa foi executada nas posturas de punho neutro, com e sem *feedback* visual, e de punho flexionado a 45°, com e sem *feedback* visual.
- Controle de força isométrica de pinça de precisão: o polegar e o indicador foram usados para aplicar 40% da força isométrica voluntária máxima do sujeito por um período de 25 segundos (3–5 N). As tarefas foram realizadas nas posturas de punho neutro, com e sem *feedback* visual, e de punho flexionado a 45°, com e sem *feedback* visual.

O *feedback* visual era removido após o participante alcançar e manter o nível de força-alvo de 40% da força máxima voluntária. Cada condição foi repetida três vezes. A variação da distância entre as polpas dos dedos (distâncias polpa-a-polpa resultante e em cada direção) e a variação da força na tarefa de pinça isométrica foram descritas pelo desvio padrão de cada série temporal e usadas como indicadores de desempenho durante as duas tarefas. A correlação entre esses desempenhos foi determinada pelo coeficiente de correlação de Pearson (r), com nível de significância de 0,05 ( *p* ) para os testes estatísticos.

## Resultados

Quanto à variabilidade do movimento de pinça em três eixos (X, Y, Z), somente o eixo X foi significativamente correlacionado ao movimento total de pinça polpa-a-polpa. Na postura neutra com os olhos abertos, identificamos uma média de força de pinça de 4,95 N e uma distância polpa-a-polpa de 12,2 cm. Quando os olhos estavam fechados na mesma postura, a força aumentou para 5,42 N, e a distância, para 13,4 cm. Na postura flexionada com os olhos abertos, a força média registrou uma diminuição para 4,68 N e a distância foi de 12,7 cm, o que indica possíveis limitações mecânicas impostas pela flexão do punho. Já na postura flexionada com os olhos fechados, a força foi de 5,05 N e a distância alcançou 13,9 cm ( Tabelas 1 2 ).

**Tabela 1:** Médias de distância polpa-a-polpa em diferentes condições experimentais

Distância polpa-a-polpa (cm)		
	Olhos abertos	Olhos fechados
Postura neutra	12,2	13,4
Postura flexionada	12,7	13,9

**Tabela 2:** Médias da força de pinça em diferentes condições experimentais

Força de pinça (N)		
	Olhos abertos	Olhos fechados
Postura neutra	4,95	5,42
Postura flexionada	4,68	5,05

O estudo revelou coeficientes de correlação variáveis, e destacou uma interação significativa entre o controle do movimento e da força. Na postura do punho neutro e sem *feedback* visual, a correlação entre a variabilidade do movimento e da força foi particularmente forte, com r = 0,77 e *p* < 0,001. Em contraste, na postura do punho flexionado com *feedback* visual, a correlação observada foi moderada, com r = 0,48 e *p* = 0,004. A ausência do *feedback* visual impactou significativamente a correlação entre a variabilidade da força e a distância polpa-a-polpa, com r = 0,73 e *p* = 0,025 na postura neutra. Adicionalmente, foi observada uma correlação significativa (r = 0,59; *p* = 0,015) entre a força sem *feedback* visual na postura neutra e com *feedback* visual na postura flexionada. As análises também apontaram correlações significativas nas diferenças de movimento e força entre as posturas neutra e flexionada, com r variando de 0,38 a 0,41 ( *p* < 0,05) para as diferenças de movimento e de 0,45 a 0,47 ( *p* < 0,01) para as diferenças de força. Esses resultados destacam as interações entre as condições experimentais e a função de pinça, e demonstram variações na correlação baseadas na postura do punho e na presença de estímulos visuais ( Tabelas 3 4 ).

**Tabela 3:** Correlações entre força e movimento nas posturas neutra e flexionada

Correlação (r)					
	DPPX	FN-OA	FN-OF	FF-OA	FF-OF
**FN-OA**	0,56				
**FN-OF**	0,73	0,83			
**FF-OA**	0,6	0,84	0,79		
**FF-OF**	0,65	0,59	0,6	0,51	

**Abreviaturas:** AO, olhos abertos; DPPX, distância polpa-a-polpa no eixo X; FF, força na postura flexionada; FN, força na postura neutra; OF, olhos fechados.

**Tabela 4:** Significância estatística nas correlações entre força e movimento nas posturas neutra e flexionada

Significância estatística (valor de *p* )					
	DPPX	FN-OA	FN-OF	FF-OA	FF-OF
**FN-OA**	0,025	1			
**FN-OF**	0,001	0	1		
**FF-OA**	0,013	0	0	1	
**FF-OF**	0,006	0,015	0,014	0,044	1

**Abreviaturas:** AO, olhos abertos; DPPX, distância polpa-a-polpa no eixo X; FF, força na postura flexionada; FN, força na postura neutra; OF, olhos fechados.

## Discussão

A pinça de preensão por oposição terminal polpa-a-polpa é a forma mais precisa de preensão, e exige oposição adequada entre polegar e indicador, integridade das polpas digitais, articulações, tendões e músculos envolvidos, especialmente o flexor profundo do segundo dedo e o flexor longo do polegar. 4 Além de executora, a mão atua como órgão somatossensorial altamente sensível, essencial para a percepção espacial, tátil e estereognóstica, pois permite reconhecer objetos sem necessidade de visão direta. 5 Neste estudo, observou-se que a postura do punho e a presença ou ausência de *feedback* visual influenciam significativamente a precisão e a força da pinça, o que demonstra a adaptabilidade dos mecanismos de controle motor manual frente a diferentes condições sensoriais e mecânicas:

**Correlações entre variáveis:** observou-se uma consistência nas correlações entre a distância e a força da pinça sob diferentes condições, o que indica que os indivíduos mantêm um padrão de controle que é influenciado tanto pelo *feedback* visual quanto pela postura do punho. Esse padrão sugere que a manipulação da pinça é adaptativa, e ajusta-se para manter a eficácia em face das mudanças sensoriais e mecânicas impostas pelas diferentes condições experimentais.

**Efeito do*feedback*visual:** a ausência de *feedback* visual resultou em um aumento tanto na força aplicada quanto na distância da pinça. Os coeficientes de correlação para força (r = 0,51 e 0,83; *p* < 0,05) e distância (r = 0,56 e 0,73; *p* < 0,05) ilustram que, na ausência de *feedback* visual, os participantes tendem a aplicar mais força e atingir uma maior distância polpa-a-polpa. Isso pode ser interpretado como uma tentativa dos indivíduos de compensar a falta de informação visual, garantindo a precisão na manipulação da pinça.

**Impacto da postura flexionada:** a flexão do punho reduziu significativamente a força de pinça, diminuindo de 4,95 N para 4,68 N com os olhos abertos (r = 0,60; *p* = 0,013). Com os olhos fechados, a força aumentou ligeiramente para 5,05 N (r = 0,51; *p* = 0,044), sugerindo uma resposta adaptativa à ausência de estímulo visual. Esses achados indicam que a flexão do punho impõe restriç ões mecânicas que podem ser parcialmente compensadas por mecanismos sensoriais ativados na ausência de estímulos visuais.

**Correlação entre variabilidade do movimento e do controle de força:** existe uma correlação notável entre a variabilidade do movimento e a variabilidade da força, com uma associação significativa observada especialmente na postura neutra sem *feedback* visual (r = 0,77; *p* < 0,001) e na postura flexionada com *feedback* visual (r = 0,48; *p* = 0,004). Isso reforça a ideia de que as variáveis de movimento e força estão interconectadas, e que essa relação é modulada tanto pela visibilidade quanto pela postura mecânica do punho.

A preensão consiste na aplicação de forças eficazes pela mão para a realização de tarefas, 6 o que exige controle preciso tanto da força quanto do movimento, e a compreensão das relações entre cinética e cinemática da pinça é essencial para interpretar alterações funcionais e patológicas da mão. Li et al. 7 investigaram os efeitos da dominância manual na variabilidade da força dos dedos durante a pinça de precisão, e observaram que a ausência de *feedback* visual aumenta a variabilidade e que a mão dominante apresenta maior precisão e coordenação. Patologias também influenciam essa função, como demonstrado por Nataraj et al., 8 que relataram que a síndrome do túnel do carpo reduz a amplitude de movimento e aumenta a variabilidade da pinça, o que afeta trajetórias, ângulos articulares e o contato digital, com prejuízo da destreza e da precisão.

É importante notar que este estudo apresenta algumas limitações. Primeiro, o tamanho da amostra foi relativamente pequeno, o que pode limitar a generalização dos resultados para outras populações. Além disso, o estudo foi realizado em condições controladas de laboratório, o que pode não refletir as condições naturais e clínicas.

A partir dos resultados, é possível inferir de forma analítica que o controle cinético e cinemático da pinça está correlacionado e não ocorre de forma independente na população estudada. A relação entre o movimento e a força na pinça sugere que esses dois processos estão interconectados, e que há uma interdependência dos mecanismos de controle na função manual.

## Conclusão

Este estudo demonstrou que o controle da função de pinça, tanto em termos de força quanto de distância entre os dedos, é significativamente influenciado pela postura do punho e pela presença de *feedback* visual. A postura neutra favoreceu maior precisão e menor variabilidade, ao passo que a flexão aumentou a variabilidade e reduziu o controle, especialmente sem *feedback* visual. Esses achados confirmam a interdependência entre os mecanismos cinético e cinemático da pinça, conforme proposto no objetivo do estudo. Assim, intervenções que considerem a postura do punho e o treinamento sensorial podem melhorar a função manual, com aplicações relevantes na prática clínica, na reabilitação e nas configurações de ergonomia. Pesquisas futuras podem desenvolver testes especializados para avaliar a pinça de precisão em patologias específicas, para validar os resultados em populações amplas e em condições reais, para aprimorar intervenções na prática clínica.
